# Metabolic syndrome and esophageal cancer risk: a systematic review and meta‑analysis

**DOI:** 10.1186/s13098-021-00627-6

**Published:** 2021-01-19

**Authors:** Jinjia Zhang, Huadong Wu, Rongying Wang

**Affiliations:** 1 Department of General Practice, Second Hospital of Hebei Medical University, Heping Western Road No. 215, Shijiazhuang, 050000 Hebei China; 2 Department of Gastrointestinal Surgery, Second Hospital of Hebei Medical University, Shijiazhuang, 050000 Hebei China

**Keywords:** Metabolic syndrome, Esophageal cancer, Meta-analysis

## Abstract

**Objective:**

Many clinical studies evaluating the relationship between metabolic syndrome and esophageal cancer yielded uncertain results. The purpose of this study is to systematically assess the relationship between metabolic syndrome and esophageal cancer.

**Methods:**

We searched clinical studies on metabolic syndrome and esophageal cancer risk in PubMed, Embase, and the Cochrane Library. Meta-analysis was conducted by RevMan 5.3 softwares.

**Results:**

A total of four cohort studies and two case–control studies met eligibility criteria and were included in the meta-analysis. Meta-analysis using a fixed-effect model indicated that MetS was related with a higher risk of EC (OR: 1.16, 95% CI 1.08–1.25). Subgroup analyses grouped by pathological types showed that MetS was related with a higher risk of EAC (OR: 1.19, 95% CI 1.10–1.28). Subgroup analyses grouped by metabolic conditions showed hyperglycemia (OR: 1.12, 95% CI 1.03–1.21),hypertension (OR: 1.23, 95% CI 1.04–1.46), obesity (OR: 1.40, 95% CI 1.22–1.60, P < 0.05) were related with a higher risk of EAC.

**Conclusions:**

Overall, our meta-analysis provides high quality evidence that metabolic syndrome was related with a higher risk of EAC. Among the individual components of the metabolic syndrome, hyperglycemia, hypertension and obesity may be the key factors.

## Introduction

Esophageal cancer (EC) represents the seventh most common malignancy worldwide. In accordance with the global cancer statistics [1]. It is the sixth leading cause of cancer-related death in the world, and the prognosis is poor, with a low 5-year survival rate [1]. Given the overall high morbidity and mortality of EC, it is critical to understand the risk factors and predisposing conditions for EC.

Metabolic syndrome (MetS) comprises a group of metabolic risk factors of cardiovascular diseases characterized by obesity, hypertension, dyslipidemia, insulin resistance [2, 3]. With the development of social economy and the change of lifestyle, the incidence of MetS increases significantly worldwide [4–6]. Chronic inflammation and oxidative stress, which are involved in carcinogenesis, are critical pathological features for MetS patients [7, 8]. Accumulating evidences show that MetS was related with significant increased colorectal [9], pancreatic [10], hepatocellular [11], breast [12], prostate [13] cancers risk.

Many clinical studies evaluating the relationship between MetS and EC have been implemented, which, however, yielded uncertain results. Drahos et al. [14] supported MetS as a risk factor for EC, whereas Lindkvist et al. [15] showed a nonsignificant relationship. Therefore, relevant clinical studies were systematically searched and meta-analyzed to assess the relationship between MetS and risk of EC.

## Methods

We carried out this meta-analysis following the guidelines of PRISMA [16] (preferred reporting items for systematic reviews and meta-analyses) and MOOSE [17] (meta-analysis of observational studies in epidemiology). Ethical approval was not necessary since this meta‑analysis used published data.

### Search strategy

We searched articles published before June 2020 using the combination of subject headings and free words in three databases (PubMed, Embase, and The Cochrane Library). This was the search strategy: (“Metabolic syndrome” OR “Metabolic Syndromes” OR “Metabolic Syndrome X” OR “Insulin Resistance Syndrome X” OR “Metabolic X Syndrome” OR “Dysmetabolic Syndrome X” OR “Reaven Syndrome X” OR “Metabolic Cardiovascular Syndrome”) AND (“Esophageal Neoplasm” OR “Esophagus Neoplasm” OR “Esophagus Neoplasms” OR “Cancer of Esophagus” OR “Cancer of the Esophagus” OR “Esophagus Cancer” OR “Esophagus Cancers” OR “Esophageal Cancer” OR “Esophageal Cancers”). The reference lists of highly relevant articles and reviews were screened for potentially eligible studies manually.

### Eligibility criteria

According to the PICOS framework (population/disease, intervention/exposure, comparison/control, outcome, and study design), studies were included if they: (1) were full-text, English-language studies; (2) were case–control, cohort or cross-sectional studied (S); (3) reported patients diagnosed with EC(P) and showed comparisons between MetS patients(I) versus those free of MetS (C); (4) reported the incidence of EC (O); (5) reported adjusted odds ratios (ORs), relative risks (RRs), or hazard ratios (HRs) and their 95% confidence intervals (CIs) directly, or sufficient data that could indirectly calculate them. Studies were excluded if they were: (1) reviews, editorials, expert opinions, comments, case reports; (2) technical, cell, animal, or cadaver experiments; or (3) abstracts or conference proceedings.

### Data extraction

Two independent reviewers (Zhang and Wu) carried out study selection, data extraction, and study quality assessment based on the predefined criteria. Any discrepancies regarding inclusion/exclusion or risk estimates were resolved by discussions between the two reviewers or judged by a third senior reviewer (Wang). We collected the following items: surname of the first author, year of publication, the country where a study population resided, study design, and characteristics of enrolled participants incorporating sample size and age, the definition of MetS, adjusted ORs and their 95% CIs, and covariates in a multivariable model.

### Risk of bias assessments

We selected the Newcastle–Ottawa Scale (NOS), a 9-star system, to assess the methodological quality of the included studies [18]. And we evaluated the quality of study in three domains: selection, comparability, and outcome or exposure. Studies rewarded more than 6 stars were rated as being high-quality.

### Statistical analysis

We used RevMan 5.3 software was employed for this meta-analysis. We adopted ORs and its 95% CIs as the effect quantities, which should be converted to their logarithms and standard errors (SEs) [19]. The I^2^ statistic and Cochran’s Q-test were evaluated to determine statistical heterogeneity among studies [20]. A random-effect model was utilized in the case of significant between-study heterogeneity (I^2^ ≥ 50% and *P* ≤ 0.1); otherwise, a fixed-effect model was employed. Sensitivity analyses was conducted by omitting one study in turn to assess its effect on the overall results [21]. When there was substantial heterogeneity, subgroup analyses were performed to seek the possible methodological and clinical heterogeneous estimates [22]. Egger regression test was adopted to judge potential publication bias using Stata 12.0 software [23].

## Results

### Results of the literature search

**Fig. 1:**
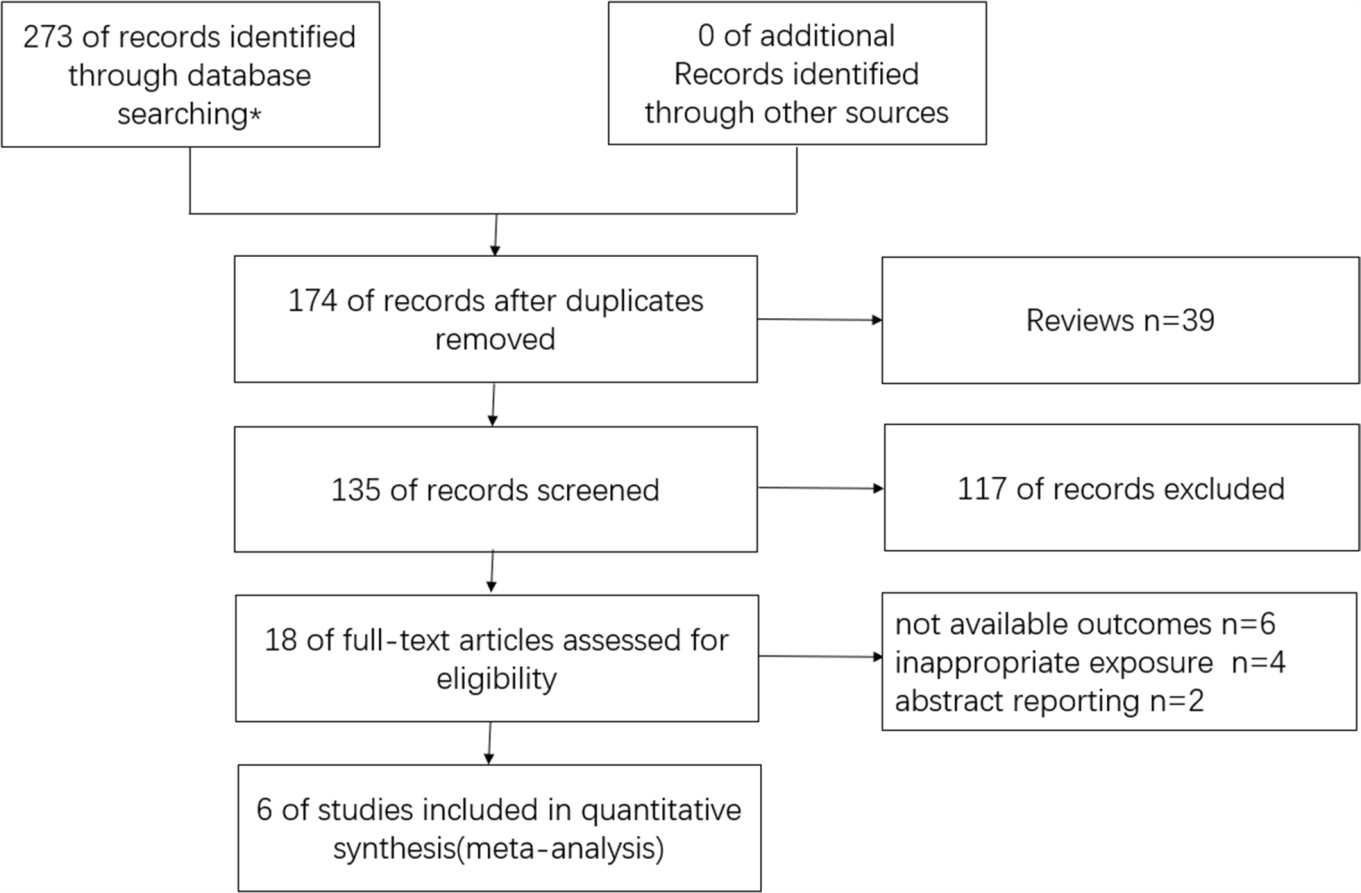
Flowchart of detailed trial selection process. Asterisk indicates the database searched and the number of studies detected are as follows: PubMed (n = 90), EMbase (n = 179). The Cochrane Library (n = 4)

During database searching, 273 relevant studies were identified from three pivotal databases and 99 duplicates were discarded. After reading the titles and abstracts, 117 ineligible studies were excluded, and the remaining 18 full texts were reviewed. Subsequently, 12 studies were excluded due to unavailable outcomes (n = 6), inappropriate exposure (n = 4), and abstract reporting (n = 2). Finally, aligning with the predefined inclusion criteria, six studies [14, 15, 24–27] involving 892,614 participants were included in our meta-analysis (Fig. 1), encompassing two case–control studies [14, 24], and four cohort studies [15, 25–27]. No additional studies from the references were added to our review. All studies were published from 2013 to 2017. Two studies [24, 25] were carried out in the United States of America(USA), one [26] in South Korea, one [27] in Norway, one [14] in the United Kingdom, and one [15] in Austria, Norway and Sweden. Table 1 listed the main characteristics of the six included studies.

**Table 1:** General characteristics of included studies

Author (year)	Country/period	Study design	Sample size (men/women)	Mean age or age range	EC assessment method	Defnition of MetS	OR (95% CI)	Adjustment
Drahos (2016) [14]	United Kingdom/1992–2012	Population based case–control study	Exp: 592 (474/118) Con: 2901 (2319/582)	Exp: 69.2 ± 11.3 Con: 68.9 ± 11.2	Medical records	NCEP-ATP III	1.01 (0.65–1.56)	Age, years of CPRD data prior to selection, sex
Drahos (2017) [24]	United States of America/2003–2009	Population based case–control study	Exp: 3167 (2481/686) Con: 15,835 (12,405/3430)	Exp: 78.0 ± 6.5 Con: 78.0 ± 6.5	ICD-9, ICD-O	NCEP-ATP III	1.16 (1.06, 1.26)	Age, sex, race, registry, smoking, history of GERD
Duggan (2013) [25]	United States of America/1995–2009	Hospital based prospective cohort	All: 392 (321/71)	All: 61.0 ± 11.5	Medical records	IDF, AHA	1.14 (0.56–2.36)	Age, sex, BMI, cigarette pack-years, regular NSAID use
Ko (2016) [26]	South Korea/2002–2013	Population based retrospective cohort	Exp: 17,989 (12,618/5371) Con: 81,576 (49,140/32,436)	≥ 20	ICD-10	≥ 3 metabolic abnormalities	0.51 (0.25–1.04)	Age, smoking status, alcohol intake, regular exercise
Lin (2015) [27]	Norway/1994–2010	Population based prospective cohort	All: 192,903 (93,058/99,845)	All: 49.5 ± 15.7	ICD-7, ICD-O-3	≥ 3 metabolic abnormalities	1.19 (0.82–1.74)	Age, sex, BMI, education, smoking status; family cancer history
Lindkvist (2014) [15]	Austria, Norway, Sweden/1972–2005	Population based prospective cohort	All: 577,259 (288,930/288,329)	All: 44.0 ± 11.7	ICD, ICD-7, ICD-O-1, ICD-O-2	5 metabolic abnormalities	1.26 (1.06–1.50)	Sex, age, smoking status

*MetS* metabolic syndrome, *NCEP-ATP III* National Cholesterol Education Program Adults Treatment Panel III, *IDF* International Diabetes Federation, *AHA* American Heart Association, *ICD* International Classification of Diseases

### Methodological quality of included studies

The quality of the included studies regarding the NOS scale was shown in Table 2, which were rewarded 6–9 stars. That meant the included studies were rated as being high-quality.

**Table 2:** Newcastle–Ottawa scale for the assessment of the quality of studies included

Cohort study	Author (year)	Selection	Comparability	Outcome	Total
1	Duggan (2013) [25]	***	*	**	6
2	Ko (2016) [26]	****	*	***	8
3	Lin (2015) [27]	****	*	**	7
4	Lindkvist (2014) [15]	****	*	***	8

Case–control study	Author (year)	Selection	Comparability	Exposure	Total
1	Drahos (2016) [14]	****	*	**	7
2	Drahos (2017) [24]	****	*	**	7

### Overall meta-analysis

**Fig. 2:**
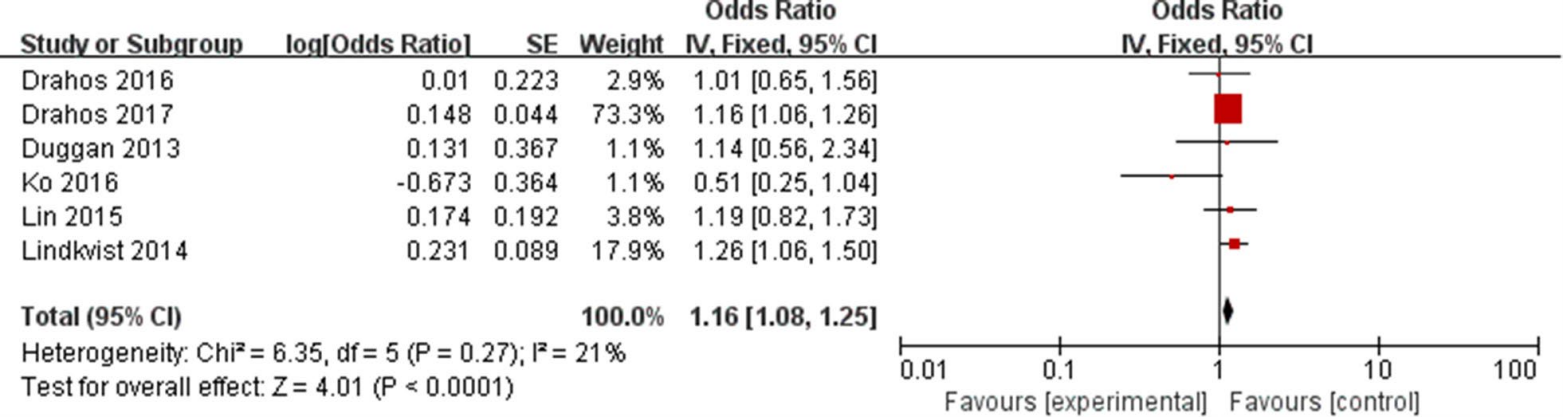
Forest plot of MetS on EC risk

**Fig. 3:**
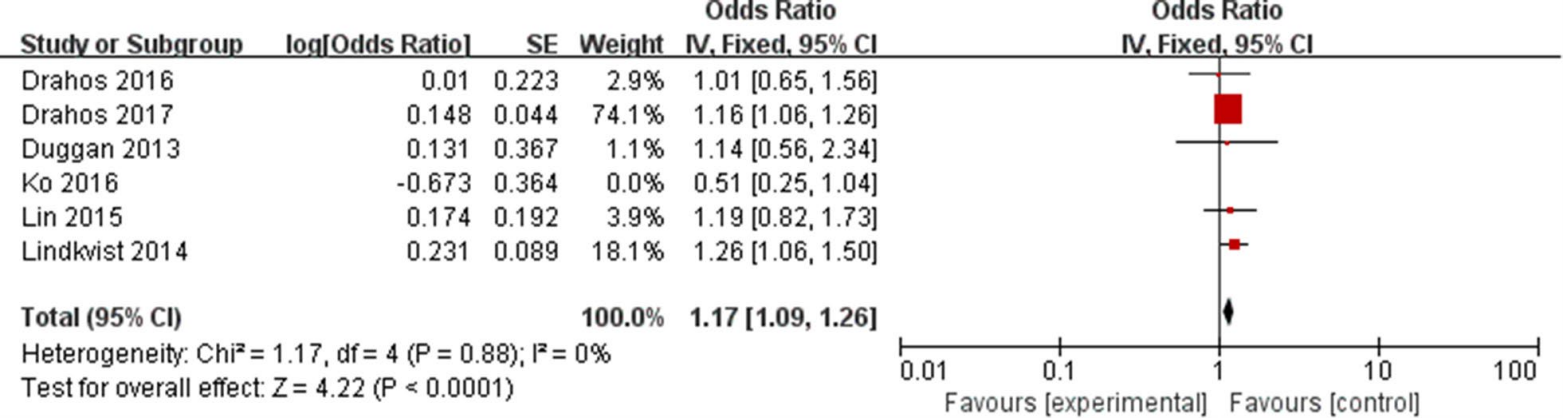
Forest plot of MetS on EC risk after removal of high heterogeneity study

The overall meta-analysis of six eligible studies [14, 15, 24–27] representing 892,614 participants was carried out using a fixed-effect model. The results showed that MetS was related with a higher EC risk (OR: 1.16, 95% CI 1.08–1.25, P < 0.05, Fig. 2), with low heterogeneity (P = 0.27, I^2^ = 21%). Sensitivity analyses demonstrated that none of these studies had a substantial effect on the effect-size correlation, suggesting robust and reliable evidence from our analysis (Table 3). When we excluded the study of Ko, I^2^ dropped from 21 to 0%. After removing this study, the result of meta-analysis based on the remaining five studies also showed that MetS was related with a higher EC risk (OR: 1.17, 95% CI 1.09–1.26, P < 0.05, Fig. 3).

**Table 3:** Results of sensitivity analysis

Omitted study	Heterogeneity		Model	Meta-analysis		
	I^2^ (%)	P		Pooled OR	95% CI of pooled OR	P
Drahos (2016)	33	0.20	Fixed-effect	1.17	1.08–1.26	< 0.0001
Drahos (2017)	37	0.18	Fixed-effect	1.17	1.02–1.35	0.03
Duggan (2013)	37	0.17	Fixed-effect	1.16	1.08–1.25	< 0.0001
Ko (2016)	0	0.88	Fixed-effect	1.17	1.09–1.26	< 0.0001
Lin (2015)	37	0.18	Fixed-effect	1.16	1.08–1.25	< 0.0001
Lindkvist (2014)	25	0.25	Fixed-effect	1.14	1.05–1.24	0.001

### Subgroup analyses

**Fig. 4:**
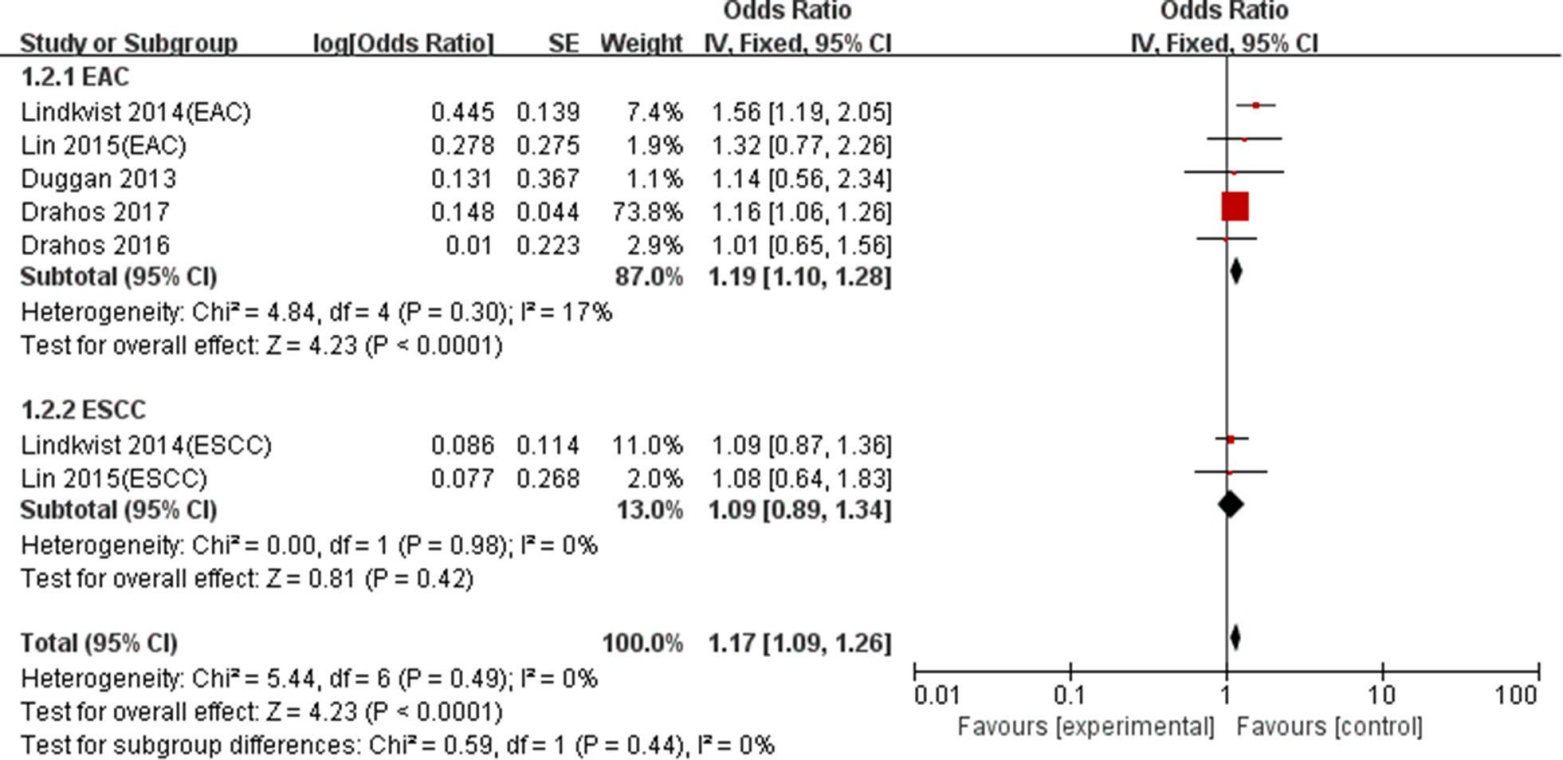
Forest plot of MetS on EC risk in regard to pathological type difference

We performed subgroup analyses based on pathological types, metabolic conditions, location and study design to explore the effect of these characteristics on the summary results (Table 4). Subgroup analyses grouped by pathological types supported that MetS was related with a higher EAC risk (OR: 1.19, 95% CI 1.10–1.28, P < 0.05), whereas showed a nonsignificant relationship between MetS and ESCC risk (OR:1.09; 95% CI 0.89–1.34; P = 0.42) (Fig. 4). Subgroup analyses grouped by metabolic conditions showed the influence of any single component of MetS on EC risk. Hyperglycemia (OR: 1.12, 95% CI 1.03–1.21, P < 0.05) and hypertension (OR: 1.23, 95% CI 1.04–1.46, P < 0.05) were related with a higher EC risk, but there were nonsignificant relationships between obesity(OR:1.15, 95%CI 0.88–1.51, P > 0.05), high cholesterol(OR: 1.05, 95% CI 0.89–1.24, P > 0.05),high triglycerides(OR: 0.96, 95% CI 0.88–1.04, P > 0.05) and EC risk.

**Table 4:** Results of subgroup analysis

Subgroup analyses	No. of studies	Heterogeneity		Model	Meta-analysis		
		I^2^ (%)	P		OR	95% CI	P
Study design							
Case–control study	2	0	0.54	Fixed-effect	1.15	1.06–1.26	0.0009
Cohort study	4	49	0.12	Fixed-effect	1.19	1.03–1.39	0.02
Location							
Asian	1	NA	NA	NA	0.51	0.25–1.04	0.06
Western	5	0	0.88	Fixed-effect	1.17	1.09–1.26	< 0.0001
Metabolic conditions							
Obesity	3	85	0.001	Random-effect	1.15	0.88–1.51	0.32
Hyperglycemia	3	0	0.80	Fixed-effect	1.12	1.03–1.21	0.006
High cholesterol	2	0	0.69	Fixed-effect	1.05	0.89–1.24	0.59
High triglycerides	2	0	0.44	Fixed-effect	0.96	0.88–1.04	0.31
Hypertension	3	67	0.05	Random-effect	1.23	1.04–1.46	0.02
Pathological types							
ESCC	2	0	0.98	Fixed-effect	1.09	0.89–1.34	0.42
EAC	5	17	0.90	Fixed-effect	1.19	1.10–1.28	< 0.0001

*ESCC* esophageal squamous cell carcinoma, *EAC* esophageal adenocarcinoma

**Fig. 5:**
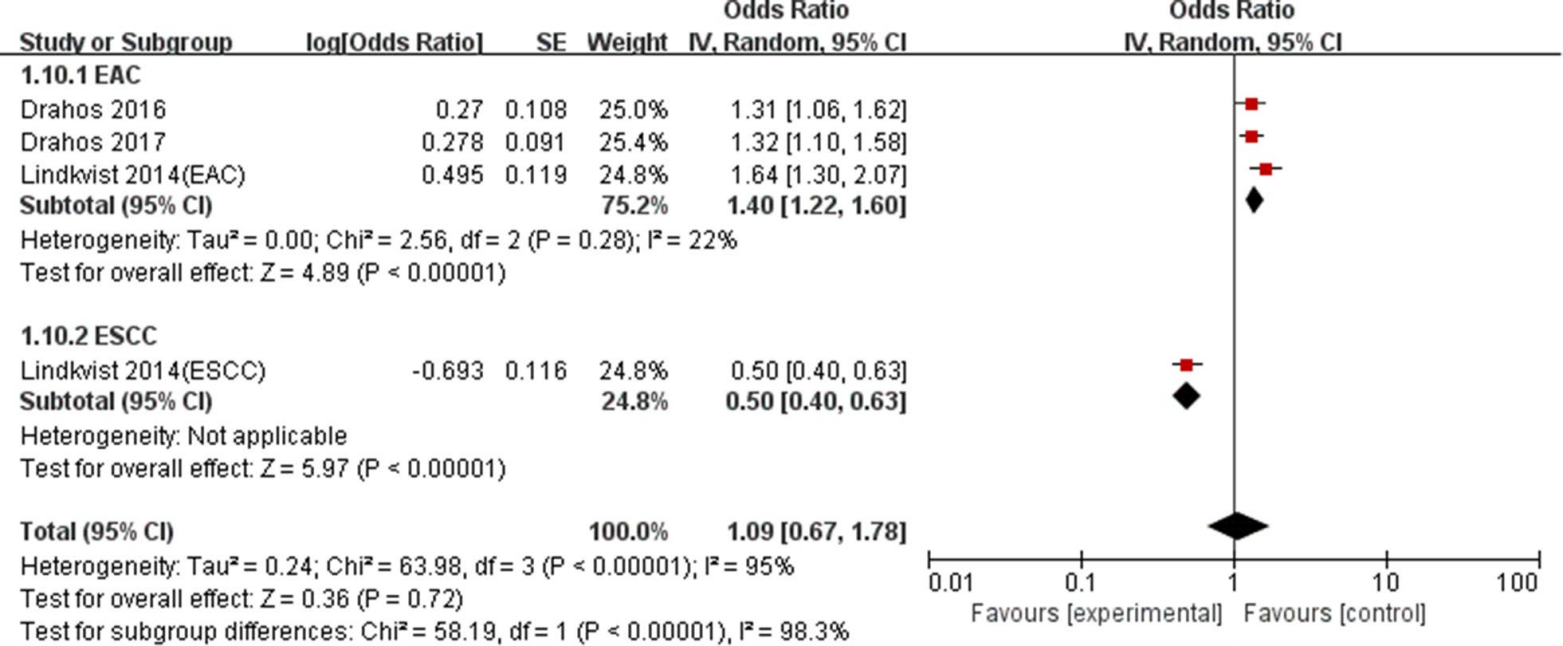
Forest plot of obesity on EC risk in regard to pathological type difference

Four independent reports from three studies [14, 15, 24] investigated the relationship between obesity and EC risk. We further analyzed this relationship according to pathological types. Meta-analysis showed a significant relationship between obesity and a higher EAC risk (OR: 1.40, 95% CI 1.22–1.60, P < 0.05), and a reduced ESCC risk (OR: 0.50; 95% CI 0.40–0.63; P < 0.05),as shown in Fig. 5.

### Publication bias

The Egger’s test provided evidence of absence of publication bias across studies (P = 0.452).

## Discussion

Our meta-analysis with a total of 892,614 participants showed that MetS was related with a higher risk of EC. Interestingly, there was a pathological type dependent difference. Among the individual components of the MetS, hyperglycemia, hypertension and obesity may be the key factors of MetS affecting the occurrence of EAC.

The relationship between MetS and EAC risk may be reasonable biologically. Epidemiologic and experimental clinical studies supports that MetS should be an critical risk factor for common tumors development [28]. MetS may favor cancer occurrence in three ways-insulin resistance, deregulation of leptin and activation of proinflammatory factor system. The first is closely related with the insulin-like growth factor 1 (IGF-1) receptor pathway.Insulin can directly or indirectly affect the occurrence of tumor through IGF-1, a powerful mitogen. In addition, due to the similar structure of insulin and IGF-1, IGF-1 can indirectly act on the heterozygous receptor to play its biological function, promote the corresponding signal transduction and cell proliferation, thus increasing the possibility of malignant tumor [29]. The second is manifested by changes in leptin and adiponectin. Leptin can promote cell proliferation through MAPK signal pathway, and promote angiogenesis and tumor occurrence by up-regulating vascular endothelial growth factor, transforming growth factor-1 and basic fibroblast growth factor [30, 31]. In contrast to leptin, adiponectin has anti-inflammatory and anti-atherosclerotic effects. Hyperleptinemia and hypoadiponectin are often found in obese people [32], which change the energy metabolism of the body and easily induce a variety of metabolic diseases, thus affecting various molecular metabolic processes of the body and increasing the risk of tumor occurrence. The third is the activation of proinflammatory factor system. The inflammatory pathway is to reduce tumor inhibition by affecting cell cycle and activating oncogene expression, thus causing tumor [33]. Meanwhile, inflammation activates the immune system, promotes the secretion and release of reactive oxygen species (Ros), and then activates the C-Jun amino terminal kinase (JNK) signal pathway to further interfere with the insulin signaling pathway [34]. Moreover, Ros products increase the risk of DNA oxidative damage by accumulating in cells, and induce DNA mutation leading to cancer [35].

The sensitivity analysis indicated that the study of Ko [26] was a major outlier, with a markedly reduced HR for EC in contrast to the other studies. However, the overall result was not affected by this study. According to the analysis of the study of Ko [26], the heterogeneity may have been due to the study location, as the study of Ko [26] was the only study from Asia. There were obvious differences in pathological types and risk factors of EC between Western and Asian countries [1, 36, 37]. In addition, in the study of Ko [26] the index number of cases was small, and ran counter to the increased rate of colorectal and other cancers in the same series. The relatively lower incidence rate meant that we need more site-specific cancer cases to clarify the effect of MetS on cancers.

Subgroup analyses showed that there was a pathological type dependent difference in the risk of EC with MetS patients. The reasons still remain to be elucidated, but there are several potential mechanisms. The occurrence of EC has obvious regional distribution. EAC is the most common type of tissue in Western countries, and obesity is the main cause [1]. Obesity, especially abdominal obesity, leads to increased intra-abdominal pressure and increases the risk of gastroesophageal reflux, which is a strong risk factor for EAC [38, 39]. Obesity is also related with increased levels of hormones such as IGF,which is known to affect cell division, cell death, and healing [40, 41].

Among the individual components of the MetS, hyperglycemia and hypertension may be the key factors of MetS affecting the occurrence of EC. The long-term hyperglycemia environment of the body will induce dysfunction of cellular respiration, leading to enhanced anaerobic respiration. The long-term hypoxia environment of the body cells is easy to induce the mutation of normal cells, which will promote the transformation of normal cells into malignant tumor cells.On the other hand, hyperglycemia will also promote the production of a large number of free radicals, further increase the risk of mutation of normal cells [42, 43]. At the same time, hyperglycemia directly promotes hyperinsulinemia and induces tumorigenesis by indirectly increasing IGF-1 function [44]. Although no relevant report has directly revealed the relationship between hypertension and EC, it has been reported that hypertension can greatly increase the risk of malignant tumors [45, 46]. In addition, basic studies have shown that calcium channel blockers can induce tumorigenesis by blocking apoptosis in the treatment of hypertension [47].

Subgroup analyses showed that there was a nonsignificant relationship between obesity and EC risk. This was different from other studies [48–50]. So we performed subgroup analysis regarding the pathological types to explore this relationship. The results showed that there was strong evidence for an relationship between obesity and an increased risk of EAC and a decreased risk of ESCC. The inverse relationship between obesity and ESCC risk may be responsible for this result.

As far as we know, this is the first meta-analysis to assess the relationship between MetS and EC. However, there are some limitations in our analysis.First, because of the observational nature of the included studies, our findings should be interpreted as exploratory. Second, assessment methods of MetS were different among included studies, which may be a source of heterogeneity. Third, there is a lack of uniformity in confounding factors under the multivariable models, which may bias the effect-size estimates. Fourth, although subgroup analyses can explain some sources of heterogeneity, we cannot identify other putative sources of heterogeneity due to insufficient data, such as sex. Finally, due to the limited availability of published studies, especially in the subgroup analyses, the results should be treated with caution, and more high-quality RCTs were needed.

## Conclusions

Overall, our meta-analysis provides high quality evidence that MetS was related with a higher risk of EAC. Among the individual components of the MetS, hyperglycemia, hypertension and obesity may be the key factors. Patients with MetS should be the focus of EAC screening and benefit from closer monitoring. In addition, behavioral interventions including controlling blood pressure and blood glucose could potentially serve as preventative measures for EAC in patients with MetS.

## References

[1] Bray F, Ferlay J, Soerjomataram I, Siegel RL, Torre LA, Jemal A (2018). Global cancer statistics 2018:globocan estimates of incidence and mortality worldwide for 36 cancers in 185 countries. CA A Cancer J Clin.

[2] Grundy SM, Cleeman JI, Merz CN, Brewer HB, Clark LT, Hunninghake DB (2004). Implications of recent clinical trials for the National Cholesterol Education Program Adult Treatment Panel III guidelines. Circulation.

[3] Alberti KG, Zimmet P, Shaw J (2006). Metabolic syndrome—a new world-wide definition. A Consensus Statement from the International Diabetes Federation. Diabet Med.

[4] Mozumdar A, Liguori G (2011). Persistent increase of prevalence of metabolic syndrome among U.S. adults: NHANES III to NHANES 1999–2006. Diabetes Care.

[5] Lim S, Shin H, Song JH, Kwak SH, Kang SM, Yoon JW (2011). Increasing prevalence of metabolic syndrome in Korea: the Korean National Health and Nutrition Examination Survey for 1998–2007. Diabetes Care.

[6] Misra A, Khurana L (2008). Obesity and the metabolic syndrome in developing countries. J Clin Endocrinol Metab.

[7] Mendonca FM, de Sousa FR, Barbosa AL, Martins SC, Araujo RL, Soares R (2015). Metabolic syndrome and risk of cancer: which link?. Metabolism.

[8] Fernandes JV, Cobucci RN, Jatoba CA, Fernandes TA, de Azevedo JW, de Araujo JM (2015). The role of the mediators of inflammation in cancer development. Pathol Oncol Res.

[9] Jinjuvadia R, Lohia P, Jinjuvadia C, Montoya S, Liangpunsakul S (2013). The association between metabolic syndrome and colorectal neoplasm: systemic review and meta-analysis. J Clin Gastroenterol.

[10] Rosato V, Tavani A, Bosetti C, Pelucchi C, Talamini R, Polesel J (2011). Metabolic syndrome and pancreatic cancer risk: a case–control study in Italy and meta-analysis. Metab Clin Exp.

[11] Li Y, Shi J, Liu X, Deng Q, Huang Y, Yang Z (2018). Metabolic syndrome relates to high risk in hepatocellular carcinoma: a meta-analysis. Discov Med.

[12] Guo M, Liu T, Li P, Wang T, Zeng C, Yang M (2019). Association between metabolic syndrome and breast cancer risk: an updated meta-analysis of follow-up studies. Front Oncol.

[13] Gacci M, Russo GI, De Nunzio C, Sebastianelli A, Salvi M, Vignozzi L (2017). Meta-analysis of metabolic syndrome and prostate cancer. Prostate Cancer Prostatic Dis.

[14] Drahos J, Li L, Jick SS, Cook MB (2016). Metabolic syndrome in relation to Barrett’s esophagus and esophageal adenocarcinoma: results from a large population-based case–control study in the Clinical Practice Research Datalink. Cancer Epidemiol.

[15] Lindkvist B, Johansen D, Stocks T, Concin H, Bjørge T, Almquist M (2014). Metabolic risk factors for esophageal squamous cell carcinoma and adenocarcinoma: a prospective study of 580,000 subjects within the Me-Can Project. BMC Cancer.

[16] Hutton B, Salanti G, Caldwell DM, Chaimani A, Schmid CH, Cameron C (2015). The PRISMA extension statement for reporting of systematic reviews incorporating network meta-analyses of health care interventions: checklist and explanations. Ann Intern Med.

[17] Stroup DF, Berlin JA, Morton SC, Olkin I, Williamson GD, Rennie D (2000). Meta-analysis of observational studies in epidemiology: a proposal for reporting. Meta-analysis of observational studies in epidemiology (MOOSE) group. JAMA.

[18] Stang A (2010). Critical evaluation of the Newcastle–Ottawa scale for the assessment of the quality of nonrandomized studies in meta-analyses. Eur J Epidemiol.

[19] Higgins J, Green S. Cochrane handbook for systematic reviews of interventions version 5.1.0. The Cochrane Collaboration; 2011. http://www.cochranehandbook.org.

[20] Higgins JPT, Thompson SG (2002). Quantifying heterogeneity in a meta-analysis. Stat Med.

[21] Patsopoulos NA, Evangelou E, Ioannidis JP (2008). Sensitivity of between-study heterogeneity in meta-analysis: proposed metrics and empirical evaluation. Int J Epidemiol.

[22] Chuang SC, Lee YC, Wu GJ, Straif K, Hashibe M (2015). Alcohol consumption and liver cancer risk: a meta-analysis. Cancer Causes Control.

[23] Egger M, Davey Smith G, Schneider M, Minder C (1997). Bias in meta-analysis detected by a simple, graphical test. BMJ.

[24] Drahos J, Ricker W, Pfeiffer RM, Cook MB (2017). Metabolic syndrome and risk of esophageal adenocarcinoma in the United States elderly: an analysis of SEER-Medicare data. Cancer.

[25] Duggan C, Onstad L, Hardikar S, Blount PL, Reid BJ, Vaughan TL (2013). Association between markers of obesity and progression from Barrett’s esophagus to esophageal adenocarcinoma. Clin Gastroenterol Hepatol.

[26] Ko S, Yoon SJ, Kim D, Kim AR, Kim EJ, Seo HY (2016). metabolic risk profile and cancer in Korean men and women. J Prev Med Public Health.

[27] Lin Y, Ness-Jensen E, Hveem K, Lagergren J, Lu Y (2015). Metabolic syndrome and esophageal and gastric cancer. Cancer Causes Control.

[28] Esposito K, Chiodini P, Colao A, Lenzi A, Giugliano D (2012). Metabolic syndrome and risk of cancer: a systematic review and meta-analysis. Diabetes Care.

[29] Pandini G, Frasca F, Mineo R, Sciacca L, Vigneri R, Belfiore A (2002). Insulin/insulin-like growth factor I hybrid receptors have different biological characteristics depending on the insulin receptor isoform involved. J Biol Chem.

[30] Braun S, Bitton-Worms K, LeRoith D (2011). The link between the metabolic syndrome and cancer. Int J Biol Sci.

[31] Lanier V, Jeffers M, Walterberger J, Anderson L, Gonzalez R (2015). Abstract 2911: leptin notch VEGFR-2 axis influences cancer stromal cell behavior. Can Res.

[32] Singh P, Sharma P, Sahakyan KR, Davison DE, Sert-Kuniyoshi FH, Romero-Corral A (2016). Differential effects of leptin on adiponectin expression with weight gain versus obesity. Int J Obes (Lond).

[33] Mendonça FM, de Sousa FR, Barbosa AL, Martin SC, Araújo RL, Soares R (2015). Metabolic syndrome and risk of cancer: which link?. Metabolism.

[34] Ray PD, Huang BW, Tsuji Y (2012). Reactive oxygen species (ROS) homeostasis and redox regulation in cellular signaling. Cell Signal.

[35] Alvarez-Cubero MJ, Entrala C, Fernandez-Rosado F, Martinez-Gonzalez LJ, Alvarez JC, Suarez A (2012). Predictive value in the analysis of RNASEL genotypes in relation to prostate cancer. Prostate Cancer Prostatic Dis.

[36] Matejcic M, Gunter MJ, Ferrari P (2017). Alcohol metabolism and oesophageal cancer: a systematic review of the evidence. Carcinogenesis.

[37] Trevellin E, Scarpa M, Carraro A, Lunardi F, Kotsafti A, Porzionato A (2015). Esophageal adenocarcinoma and obesity:peritumoral adipose tissue plays a role in lymph node invasion. Oncotarget.

[38] Fass R (2008). The pathophysiological mechanisms of GERD in the obese patient. Dig Dis Sci.

[39] Fornari F, Madalosso CAS, Farre R, Gurski RR, Thiesen V, Callegari-Jacques SM (2010). The role of gastro-oesophageal pressure gradient and sliding hiatal hernia on pathological gastro-oesophageal reflux in severely obese patients. Eur J Gastroenterol Hepatol.

[40] Dieudonne MN, Bussiere M, Dos Santos E, Leneveu MC, Giudicelli Y, Pecquery R (2006). Adiponectin mediates antiproliferative and apoptotic responses in human MCF7 breast cancer cells. Biochem Biophys Res Commun.

[41] Renehan AG, Zwahlen M, Minder C, O’Dwyer ST, Shalet SM, Egger M (2004). Insulin-like growth factor (IGF)-I, IGF binding protein-3, and cancer risk: systematic review and meta-gegression analysis. Lancet.

[42] Chang CK, Ulrich CM (2003). Hyperinsulinaemia and hyperglycaemia: possible risk factors of colorectal cancer among diabetic patients. Diabetologia.

[43] Brownlee M (2005). The pathobiology of diabetic complications a unifying mechanism. Diabetes.

[44] Alberti KG, Zimmet P, Shaw J, IDF Epidemiology Task Force Consensus Group (2005). The metabolic syndrome—a new worldwide definition. Lancet.

[45] Milan A, Puglisi E, Ferrari L, Bruno G, Losano I, Veglio F (2014). Arterial hypertension and cancer. Int J Cancer.

[46] Stocks T, Van Hemelrijck M, Manjer J, Bjørge T, Ulmer H, Hallmans G (2012). Blood pressure and risk of cancer incidence and mortality in the metabolic syndrome and cancer project. Hypertension.

[47] Fryzek JP, Poulsen AH, Johnsen SP, McLaughlin JK, Sørensen HT, Friis S (2005). A cohort study of antihypertensive treatments and risk of renal cell cancer. Br J Cancer.

[48] Corley DA, Kubo A, Zhao W (2008). Abdominal obesity and the risk of esophageal and gastric cardia carcinomas. Cancer Epidemiol Biomarkers Prev.

[49] MacInnis RJ, English DR, Hopper JL, Giles GG (2006). Body size and composition and the risk of gastric and oesophageal adenocarcinoma. Int J Cancer.

[50] Steffen A, Schulze MB, Pischon T, Dietrich T, Molina E, Chirlaque MD (2009). Anthropometry and esophageal cancer risk in the European Prospective Investigation into cancer and nutrition. Cancer Epidemiol Biomarkers Prev.

